# An adolescent girl with premature ovarian failure, Graves’ disease, and chronic urticaria: a case report

**DOI:** 10.1186/s13256-020-02491-w

**Published:** 2020-10-11

**Authors:** Danhong Lin, Huibiao Quan, Kaining Chen, Lu Lin, Leweihua Lin, Qun Ji

**Affiliations:** Department of Endocrinology, Hainan General Hospital, Hainan Affiliated Hospital of Hainan Medical University, No. 19, Xiuhua Road, Xiuying District, Haikou City, Hainan Province China

**Keywords:** Premature ovarian failure, Graves’ disease, Chronic urticaria, Autoimmune polyglandular syndrome

## Abstract

**Background:**

Premature ovarian failure is characterized by amenorrhea, hypoestrogenism, and hypergonadotropinism, and occurs in women under 40 years of age. The prevalence of premature ovarian failure in women younger than 20 years of age is only 0.01%. Immune disorders are one of the causes of premature ovarian failure. Graves’ disease and chronic urticaria are also associated with immune disorders.

**Case presentation:**

We report a case of a 15-year-old Han Chinese girl with premature ovarian failure complicated by Graves’ disease and chronic urticaria. She experienced menarche at 13 years of age and presented with amenorrhea after 7 months of irregular menstruation. Laboratory examinations indicated hypoestrogenism and hypergonadotropinism. Ultrasound imaging revealed that her uterus and ovaries were small in size. Gene and antibody tests related to premature ovarian failure returned negative results. Both thyroid peroxidase autoantibody and thyrotropin receptor antibody were positive.

After reviewing the literature on the relationship between these three diseases and immune disorders, our patient was diagnosed as having atypical autoimmune polyglandular syndrome. After taking small doses of estrogen for 6 months, the size of her uterus increased, and her psychological anxiety was relieved.

**Conclusions:**

We report a case of an unusual association of premature ovarian failure, Graves’ disease, and chronic urticaria. This case presents an atypical combination of adolescent autoimmune polyglandular syndrome, which is worthy of the attention of clinicians and presents an important lesson for them. Our case highlights that premature ovarian failure in adolescents requires long-term follow-up and medical treatment as well as psychological counselling.

## Background

The concept of premature ovarian failure (POF) was introduced in 1960. Its diagnostic criteria include amenorrhea for more than 4 months before the age of 40, excluding a period of pregnancy, and at least two blood draws (obtained at least 1 month apart) during the follicular phase (days 3–6 of the menstrual cycle) indicating serum follicle-stimulating hormone (FSH) > 40 U/L and estradiol < 30 ng/L. POF is one of the causes of secondary amenorrhea, and the ovaries as well as the thyroid are the targets of immune attack. Chronic urticaria is also associated with immune disorders.

## Case presentation

A 15-year-old Han Chinese girl presented to our department in June 2019 with the chief complaint of amenorrhea for 2 years. She experienced menarche more than 2 years ago with a menstrual cycle of 18–21 days and menstruation lasting 6–7 days with normal menstrual flow. However, the menstruation ceased 7 months after menarche with no obvious cause. Her last menstruation was on June 26, 2017. Her past medical history revealed recurrent attacks of urticaria 6 years ago, which were attributed to allergies to abalone, milk, soy, and eggs. She was diagnosed as having Graves’ disease 4 years ago. She was treated with thiamazole, which was discontinued after more than 2 years of treatment and her thyroid function returned to normal with monitoring. Her personal history revealed that she had a full-term birth and a birth weight of 2.85 kg. The developmental milestones of raising her head, turning over, sitting up, crawling, and walking were achieved at normal times, and her academic performance was good. Her mother was 42-years old and had regular menstruation; our patient’s maternal grandmother experienced menopause at the age of 50.

A physical examination revealed: a body temperature of 36.2 °C; pulse, 63 beats/minute; respiratory rate, 20 breaths/minute; blood pressure, 93/60 mmHg; height, 161.5 cm; body weight, 47 kg; upper body height 80.5 cm; lower body height 81 cm; and arm span 161.5 cm. The appearance of her vulva was normal, with Tanner II breast development and Tanner I pubic hair development. Cardiopulmonary and abdominal examinations revealed no abnormalities, and there were no malformations such as webbed neck, cubitus valgus, or shortened metacarpals. Supplemental examination results of adrenocorticotropic hormone, cortisol, sex hormones, and thyroid hormones are shown in Tables 1, 2 and 3. The results of blood, urine, and fecal tests were normal; the results of electrolytes test, liver function test, and kidney function test were normal. Ultrasound examinations of her breasts, thyroid, and abdomen revealed no abnormalities. Her lumbar spine bone density T-score was − 1.5. Her growth hormone level at 1 hour of deep sleep was 2.19 ng/mL (reference range, 0.123–8.05) and parathyroid hormone level was 30.5 pg/mL (reference range, 15–68.3). Her anti-Müllerian hormone level was < 0.06 ng/mL (reference range, 2.80–6.3). Serum anti-ovarian antibody, antinuclear antibody, rheumatoid factor, antineutrophil cytoplasmic antibodies, and extractable nuclear antigens polypeptide titers were negative. A transrectal color Doppler ultrasound revealed the size of her uterus to be 19 × 15 × 22 mm, with a small volume and an intimal thickness of approximately 3 mm. The sizes of her left and right ovaries were 16 × 7 × 8 mm and 18 × 7 × 9 mm, respectively, indicating that both ovaries were small. A 3.7 × 3.3 mm sized follicle was visible in her left ovary. Magnetic resonance imaging of her pituitary gland indicated no abnormalities. Her bone age was approximately 15–16 years. Her chromosomal type was 46,XX as per the karyotype analysis of 50 cells. Whole-exome genetic testing did not reveal mutations associated with amenorrhea. Gene copy number testing revealed no obvious pathological copy number variation (CNV).

**Table 1:** Adrenocorticotropic hormone and cortisol values of the patient

	Cortisol (nmol/l)			ACTH (pg/ml)		
	08:00 a.m.	16:00 p.m.	00:00 a.m.	08:00 a.m.	16:00 p.m.	00:00 a.m.
Patient	421	166	30	58.62	18.20	6.5
Reference range	101–535.7	79–477.8	79–477.8	7.2–63.3	7.2–63.3	7.2–63.3

*ACTH* adrenocorticotropic hormone

**Table 2:** Values of different sex hormones in the patient

	LH (IU/L)	FSH (IU/L)	E2 (ng/L)	PRL (mIU/L)	Prog (nmol/L)	Testo (nmol/L)
First test	30.86	87.27	15.8	268.8	1.23	0.67
Second test (1 month later)	30.34	88.16	< 10	190.2	0.82	0.76
Reference range	0.56–14.0	1.38–5.47	21–312	108–557	3.82–50.56	0.43–2.06

*E2* estradiol, *FSH* follicle-stimulating hormone, *LH* luteinizing hormone, *PRL* prolactin, *Prog* progesterone, *Testo* testosterone

**Table 3:** Values of thyroid hormones in the patient

	TT3 (nmol/l)	TT4 (nmol/l)	FT3 (pmol/l)	FT4 (pmol/l)	TSH (mIU/L)	TPOAb (IU/ml)	TRAb (IU/L)
Patient	1.54	94.12	4.24	11.85	0.809	> 1000	5.01
Reference range	0.86–2.44	62.68–150.8	2.63–5.7	9.63–18.33	0.35–4.94	< 5.61	≤ 1.75

*FT3* free triiodothyronine, *FT4* free thyroxine, *TPOAb* thyroid peroxidase autoantibody, *TRAb* thyrotropin receptor antibody, *TSH* thyroid-stimulating hormone, *TT3* triiodothyronine, *TT4* thyroxine

After learning about the disease, our patient suffered from anxiety and insomnia. Psychologists counselled and reassured her that treatment could lead to improvement. Estazolam was prescribed as a sleep aid for a short period, together with 0.5 mg estradiol once daily, calcium, and vitamin D replacement. Follow-up and ultrasound re-examination after 6 months of treatment revealed that her uterus had increased in size to 24 × 16 × 25 mm. There was no significant change in the bilateral ovarian size, and no follicles were found. Further examination showed Tanner III breast development and Tanner I pubic hair. Her lumbar spine bone density T-score was − 1.0. The treatment had had a favorable effect and resulted in improving our patient’s psychological anxiety.

## Discussion and conclusions

The patient in our case was a 15-year-old girl with normal height, intelligence, and physical appearance. She presented with short-term irregular menstruation followed by amenorrhea, which was considered secondary amenorrhea. Laboratory examinations indicated hypergonadotropic hypoestrogenic amenorrhea and small uterine and small ovarian volumes. Turner syndrome (TS), 46,XX pure gonadal dysgenesis, and POF should be considered in cases of secondary hypergonadotropic amenorrhea.

Typically, TS is characterized by incomplete development of the secondary sexual characteristics, primary hypergonadotropic amenorrhea, short stature, physical malformations, rudimentary uterus, and streak ovaries. Approximately half of the patients with TS are haploid for the X chromosome (45,XO), 20–30% are chimeric (45,XO/46,XX), and the rest have structural abnormalities of the X chromosome [1]. Peripheral blood karyotype analysis is the gold standard for TS diagnosis. Since the karyotype analysis of 50 cells from our patient indicated no abnormalities and she did not show the characteristic TS appearance, TS was ruled out. The condition of 46,XX pure gonadal dysgenesis is either inherited by an autosomal recessive pattern or may occur sporadically. It presents as primary amenorrhea and an immature vulva, and is commonly accompanied by sensorineural deafness. In some conditions, the clinical presentation, gonadal development, and changes in sex hormones are similar to those presented in TS. However, it is caused by mutations in the FSH receptor gene in some patients with 46,XX karyotype. Our patient had secondary amenorrhea, and there were no abnormalities detected by whole-exome genetic testing or CNV testing; hence, 46,XX pure gonadal dysgenesis was not considered.

### POF and immune disorders

The relevant examinations conducted on this patient indicated a diagnosis of POF, even though POF at such a young age is rare. Common causes of POF include genetic mutations, chromosomal abnormalities, immune disorders, iatrogenic factors, and environmental injury. According to the literature, the cause of POF could be determined only in 10–15% of patients [2]. The incidence of POF is 1% among women aged under 40 years, 0.1% in those under 30 years of age, and 0.01% in those under 20 years of age (excluding patients with TS or other known chromosomal abnormalities). In cases of amenorrhea due to abnormal ovarian function in adolescents, most cases are of primary amenorrhea and only 13% are cases of secondary amenorrhea [3]. Our patient presented a case of secondary amenorrhea, which is relatively rare.

Approximately 5–30% of patients with POF have a concomitant autoimmune disease such as autoimmune thyroid disease, Crohn’s disease, or systemic lupus erythematosus. The ovaries are often targeted by the immune system, and the mechanism of immune injury involves an increase in antibody-producing B lymphocytes, decreases in the CD4^+^/CD8^+^ lymphocyte ratio, and decreases in the number and activity of natural killer cells. Anti-ovarian antibodies can be detected in some patients with POF. In addition, pathological examination can confirm lymphocytic infiltration of the follicles and ovarian interstitial tissue at all levels [4].

Whole-exome genetic testing and CNV testing of our patient revealed no abnormalities, suggesting that the cause of POF was related to autoimmunity, despite the absence of ovarian antibodies. POF is considered the end stage of the disease. When a woman is diagnosed as having POF, it suggests that the follicle supply has been depleted, and this may also deplete the target antigen of autoimmune attack. Therefore, the causality of autoimmunity in POF is difficult to track [5]. The diversity of ovarian antigens and the differences among various laboratory testing methods indicate that ovarian antibodies are a poor reflection of ovarian failure due to immune disorders [6]. Anti-ovarian antibodies are detected in only 30–67% of patients with POF.

### Chronic urticaria, immune disorders, and thyroid disease

Urticaria lasting for over 6 weeks can be categorized as chronic urticaria. It manifest as hives and erythema, and its incidence in the general population is 0.5–5% [7]. The main mechanism in chronic urticaria is the release of vasoactive transmitters, such as histamine, via mast cells and basophils degranulation. In addition, immunoglobulin E (IgE) receptor (FcεR1α) autoantibodies, Thl and Th2 cell imbalance, and abnormal leukotrienes are also part of the pathogenic mechanism of urticaria [8]. Many autoimmune diseases, including immune thyroid disease, systemic lupus erythematosus, and polymyositis, are associated with chronic urticaria [9].

Even in patients with chronic urticaria with normal thyroid function, the positivity rates of thyroglobulin antibodies and thyroid peroxidase antibodies (TPOAb) are higher than those of healthy controls [10]. Schmetzer *et al.* reported that over 200 IgE-specific antigens were detected in patients with chronic urticaria, and interleukin-24 was present in all of them, but was absent in the control group [11]. Both men and women with chronic urticaria were more likely (7 and 23 times, respectively) to develop hypothyroidism than the control group. In 80% of these patients, thyroid disease was diagnosed within 10 years of urticaria diagnosis [12]. Similarly, our patient developed urticaria first and subsequently developed Graves’ disease.

### Autoimmune polyglandular syndrome (APS)

The above discussions show that the POF, Graves’ disease, and chronic urticaria observed in our patient are all autoimmune-related. The three main hallmarks of APS-1 are Addison disease, hypoparathyroidism, and mucocutaneous candidiasis. APS-1 is associated with a mutation in the *AIRE* gene, which regulates self-tolerance to T cell attack in the thymus; thus, mutation of this gene causes T cells to attack themselves. Approximately 45–60% of women with this genetic mutation have POF, but only approximately 2% of men have testicular failure [4]. APS-2 is associated with abnormalities of human leukocyte antigen (HLA) alleles. The major histocompatibility complex encoded by HLA can affect immune tolerance at various levels. The three main hallmarks of APS-2 are Addison disease, autoimmune thyroid disease, and type 1 diabetes. Its minor hallmarks include immune ovarian failure, chronic atrophic gastritis, and lymphocytic hypophysitis. Some researchers have also proposed other forms of APS, namely APS-3 and APS-4. APS-3 is an autoimmune thyroid disease with one or more concomitant autoimmune diseases but does not include Addison disease. APS-4 refers to a combination of autoimmune diseases that do not belong to the three aforementioned categories. Currently, the two-type classification method is mostly used, that is, both APS-3 and APS-4 are classified as APS-2.

Ruggeri *et al.* reported the case of a 36-year-old woman with Graves’ disease, POF, and chronic urticaria. Her laboratory investigations revealed abnormal HLA haplotypes. Although her serum anti-ovarian antibody titer was negative, the authors concluded the presence of autoimmune ovarian failure and a diagnosis of APS [13].

Our patient had a history of Graves’ disease and had been taking thiamazole for 2 years. She tested positive for TPOAb and thyrotropin receptor antibody, leading to a diagnosis of autoimmune thyroid disease. Furthermore, she was suspected to have immune POF and chronic urticaria, leading to a diagnosis of atypical APS-2. Hence, close follow-up is required to detect other hallmarks such as Addison disease and type 1 diabetes as early as possible. The time of appearance of different APS hallmarks can span from several years to several decades. Studies have shown that POF may appear 8–14 years prior to Addison disease [14].

### Treatment of POF in adolescents

Estrogen deficiency in adolescents leads to underdeveloped secondary sexual characteristics, which can lead to a range of mental health problems, as well as an increased risk of cardiovascular disease and osteoporosis. Patients with POF have a higher risk of osteoporosis than post-menopausal women [15]. In adolescent patients with POF, administration of estrogen in small doses and gradually increasing the dosage is an alternative treatment for stimulating normal development, along with the basic treatment of calcium and vitamin D administration. DiVasta and Gordon recommended administration of 1/8^th^ to 1/10th of the adult estrogen dose before the patient’s height reaches the expected height in order to avoid any negative effects on the final adult height [16]. A randomized controlled trial by Shah *et al.* showed that transdermal estrogen administration was more effective than oral administration [17]. The recommended starting dose is 0.0125 mg/day, which is doubled every 6 months to a maximum dose of 0.05 mg/day, and progesterone is added after 18 months of treatment. The treatment should transition to complete sequential estrogen and progesterone hormone therapy once induction of puberty and breast development is complete. In women with POF undergoing regular hormone replacement therapy, bone mineral density significantly increased after 3 years of treatment, with no difference from the control group with normal ovarian function [18]. After treatment, the uterus and breasts of this patient were more developed than before. Her bone mineral density increased, and she was relieved of psychological anxiety.

### Summary

We presented a case of atypical combination of adolescent APS. Adolescent POF is rare and warrants attention and research by clinicians. We intend to follow-up with our patient over a long period and adjust and optimize the diagnosis and treatment plan according to any changes in her condition and needs.

Moreover, special attention must be given to the impact of POF in adolescents on their psychological health. Adolescents diagnosed as having POF often describe the experience using words such as “confusion, depression, anxiety, emptiness, loss and shock” [19]. Our patient also experienced anxiety after learning of the possibility of infertility, which gradually eased with long-term psychological counselling by physicians and family members.

## Data Availability

Not applicable.

## Abbreviations

POF: Premature ovarian failure
APS: Autoimmune polyglandular syndrome
FSH: Follicle-stimulating hormone
IgE: Immunoglobulin E
CNV: Copy number variation
TS: Turner syndrome
TPOAb: Thyroid peroxidase antibodies
HLA: Human leukocyte antigen
